# Medical Students’ Perception Toward Using AI in Medical Education in the Kurdistan Region, Iraq: A Cross-Sectional Study

**DOI:** 10.7759/cureus.70545

**Published:** 2024-09-30

**Authors:** Dawan J Hawezy, Kochr A Mahmood, Gasheen A Hawezy, Govand S Sadraldeen, Saddon T Ahmad

**Affiliations:** 1 Surgery, Koya University, Koya, IRQ; 2 Medicine, Koya University, Koya, IRQ

**Keywords:** ai, kurdistan region, medical education, medical students, perception

## Abstract

Background and aim

AI is revolutionizing medical education by offering innovative tools and simulations that augment traditional teaching methods. This study explored the perceptions and expectations of medical students in the Kurdistan region, Iraq, regarding AI integration in medical education.

Methods

A cross-sectional online survey collected data from 224 medical students over four months. A descriptive analysis was conducted to present the student's attitudes.

Results

In total, 224 medical students responded to the online survey. The majority of them were female (n=129; 57.6%), while 95 were male (42.4%). Additionally, most of the participants were in stage 4 (54 (24.1%); stage 1, 48 (21.4%); and stage 2, 43 (19.2%). In terms of measuring students’ perceptions of AI integration in medical education, 186 (83%) of the students wanted to use smartphones and tablets, and 38 (17%) of them reported wanting hard copies. In addition, 112 (50%) of the medical students considered themselves experts in using AI and 98 (43.8%) did not know exactly what AI was used; however, only a few of them (6.3%) did not use AI. Few patients reported using Manikins instead of real patients (42 (18.8%)), while 140 (62.5%) reported that they could be used but not an alternative.

Conclusion

While many agree that digital tools and simulations are useful teaching tools, they are frequently viewed as adjunctive approaches. Better integration and training are required for the infrequent use of AI tools in medical education.

## Introduction

AI is a universal term that refers to technology that enables computers to mimic human intellect [1]. AI, an important branch of computer science, was formally introduced in 1956 and was first used in a conference at Dartmouth College in the U.S. It is now called one of the three leading technologies in the world [2]. Current developments in technologies such as computers and informatics have made possible the use of AI and, in particular, deep learning systems in the field of medicine [3].

Healthcare providers are responsible for confirming that AI applications provide useful technology in support of patient care. For this reason, obtaining adequate knowledge and skills regarding AI applications in medicine is important for medical students, who may even have to use applications that did not exist during their education [4]. Thus, the World Medical Association promoted a review of medical curricula and educational opportunities for patients, physicians, medical students, health administrators, and other healthcare professionals to foster a better understanding of several aspects of AI in healthcare, both positive and negative aspects [5]. Technologies such as AI are needed to enable healthcare professionals to effectively apply this knowledge to practice medicine [6]. As medicine and the delivery of healthcare age increase, the need for competent human-machine interactions for the use of data to aid clinical decision-making will increase [7]. Medical students need to be sufficiently proficient in understanding the basic concepts of how AI functions and its advantages in improving expenses, increasing quality, and easing access to healthcare [8,9]. Similarly, students must be educated about the shortfalls of AI, such as transparency and liability [10]. Finally, overlooking a technology that will be transformative for the foreseeable future would place medical students at a disadvantage.

Simulation is a technique for practice and learning that can be applied to many different disciplines and types of trainees. It is a technique (not a technology) to replace and amplify real experiences with guided experiences, often “immersive” in nature, that evoke or replicate substantial aspects of the real world in a fully interactive fashion. “Immersive” here implies that participants are immersed in a task or setting as if it were the real world [11]. Previously in Palestine, it was confirmed that medical students had a lack of access and knowledge regarding using AI in their field [12].

The primary change made to the educational system, including medical education, was e-learning. The present study in the Kurdistan region will offer insights into the potential range of outcomes for the effectiveness of medical education. Until the educational system transitioned to a hybrid system, e-learning showed successful outcomes in sustaining learning [13].

The aim of this study was to determine the perceptions and future expectations of medical students regarding the use of AI in medical education.

## Materials and methods

Study design

This study employed a cross-sectional online survey design to gather data on the perceptions and expectations of medical students regarding the integration of AI in medical education. The survey was administered over a period of four months, from February 2024 to June 2024.

Participants

The participants included medical students from various medical schools and institutions in the Kurdistan region, Iraq. The inclusion criteria were medical students currently enrolled in an undergraduate or postgraduate medical program, all the medical students in the Kurdistan region at any university, and patients who provided informed consent to participate in the study.

Sampling method

A nonprobability sampling method, specifically a convenience sampling method, was used to recruit participants. Invitations to participate in the survey were distributed through the following: email lists of medical universities, social media platforms (e.g., Twitter, WhatsApp groups, Facebook groups, Viber Groups, and Telegram groups related to medical education), and online forums and communities for medical professionals and students.

Survey tool

A self-administered questionnaire was developed based on a comprehensive review of the literature on AI in medical education [12]. The survey included closed-ended questions and was structured.

Demographic Characteristics

Demographic characteristics include age, sex, name of the university, and stage of the study.

Perceptions

Questions measuring perceptions of AI integration in medical education were included. The four questions related to the use of AI during medical education included “use of manikins instead of real patients,” “use of simulation instead of laboratory,” “use of smart applications instead of hospital visits,” and “use of anemia simulation instead of real cadaver.” All the questions were answered with four options (good choice, can be used but not as an alternative, bad choice, and never accepted). Another three questions were asked about the use of AI tools: preparing lecturers and seminars, filling out logbooks and case series, and preparing reports and articles. All of these questions were answered on a Likert scale ranging from never to always.

Students were asked about their perceptions of AI in the near future in the medical field, and five options were given for selection; they had the opportunity to select more than one option.

The survey was created and administered using an online survey platform (Google Forms). The survey link was distributed through the channels as described previously, and participants were given a period of eight weeks to complete the survey. The reminders were sent two weeks and one week before the survey closing date to maximize response rates.

Data analysis

The first four questions were answered as follows: “good choice," "can be used but not as an alternative,” “bad choice,” or “never accepted,” numbered from 3 to zero. The Likert scale questions were coded from -2 to 2.

Quantitative data from closed-ended questions were analyzed using descriptive and inferential statistics. Descriptive statistics such as frequencies, percentages, means, and standard deviations were calculated for demographic variables and Likert scale responses. Inferential statistics were used to analyze the data using SPSS version 27 (IBM SPSS Statistics, Armonk, NY).

Ethical considerations

Ethical approval for the study was obtained from the scientific and research board of the faculty of medicine at Koya University (3/15/23-1-2024). Informed consent was obtained from all participants, and they were assured of the confidentiality and anonymity of their responses. Participation was voluntary, and participants could withdraw from the study at any time without any consequences.

## Results

In total, 224 medical students responded to the online survey. The mean age was 19.9±1.98. The majority of them were female (n=129; 57.6%), while 95 were male (42.4%). Additionally, most of the participants were in stage 4 (54 (24.1%); stage 1, 48 (21.4%); and stage 2, 43 (19.2%). In addition, the majority of the medical students who answered the questions were from Koya University (130; 58%), and 94 were from other universities (42%). Table 1 includes the details.

**Table 1:** Demographic characteristics of the participants

Variables	Frequency	Percentage
Mean age ± SD	19.9±1.98	
Gender		
Female	129	57.6
Male	95	42.4
Stage in college		
Stage 1	48	21.4
Stage 2	43	19.2
Stage 3	37	16.5
Stage 4	54	24.1
Stage 5	19	8.5
Stage 6	23	10.3
Which university do you study?		
Koya University	130	58
Other universities	94	42

In terms of measuring students’ perceptions of AI integration in medical education, 186 (83%) of the students wanted to use smartphones and tablets, and 38 (17%) of them reported wanting hard copies. In addition, 112 (50%) of the medical students considered themselves experts in using AI and 98 (43.8%) did not know exactly what AI was used; however, only a few of them (6.3%) did not use AI. Few patients reported using Manikins instead of real patients (42 [18.8%)), while 140 (62.5%) reported that they could be used but not as an alternative. In addition, 86 (38.4%) participants answered poorly in the simulation test, but 91 (40.6%)) answered can be used but not as an alternative. In terms of smart applications, instead of hospital visits, 107 (48.8%) selected bad choice, while 49 (21.9%) selected could be used but not as an alternative. In anatomy simulation instead of a real cadaver, 56 (25%) answered it is a bad choice, while nearly half of them (100 (44.6%)) stated that it can be used but not as an alternative.

Overall, there is a tendency toward accepting technology and simulations as supplementary tools but not as complete replacements for traditional methods (Table 2).

**Table 2:** Medical students’ perception of AI integration in medical education

Questions	Frequency	Percentage
Are you a smartphone and tablet user or do you prefer hard copies of lectures during studying?		
Want hard copies	38	17
Want to use smartphones and tablets	186	83
Have you ever heard or read about AI?		
No	14	6.3
Yes, but I don’t know what is it exactly	98	43.8
Yes, and I would consider myself as an expert	112	50
During college study, do you use manikins instead of real patients?		
Bad choice	42	18.8
Can be used but not as an alternative	140	62.5
Good choice	32	14.3
Never accepted	10	4.5
During college study, do you use simulation instead of laboratory?		
Bad choice	86	38.4
Can be used but not as an alternative	91	40.6
Good choice	28	12.5
Never accepted	19	8.5
During college study, do you use smart applications instead of hospital visits?		
Bad choice	107	48.8
Can be used but not as an alternative	49	21.9
Good choice	14	6.3
Never accepted	54	24.1
During college study, do you use anatomy simulation instead of real cadavers?		
Bad choice	56	25
Can be used but not as an alternative	100	44.6
Good choice	32	14.3
Never accepted	36	16.1

**Figure 1:**
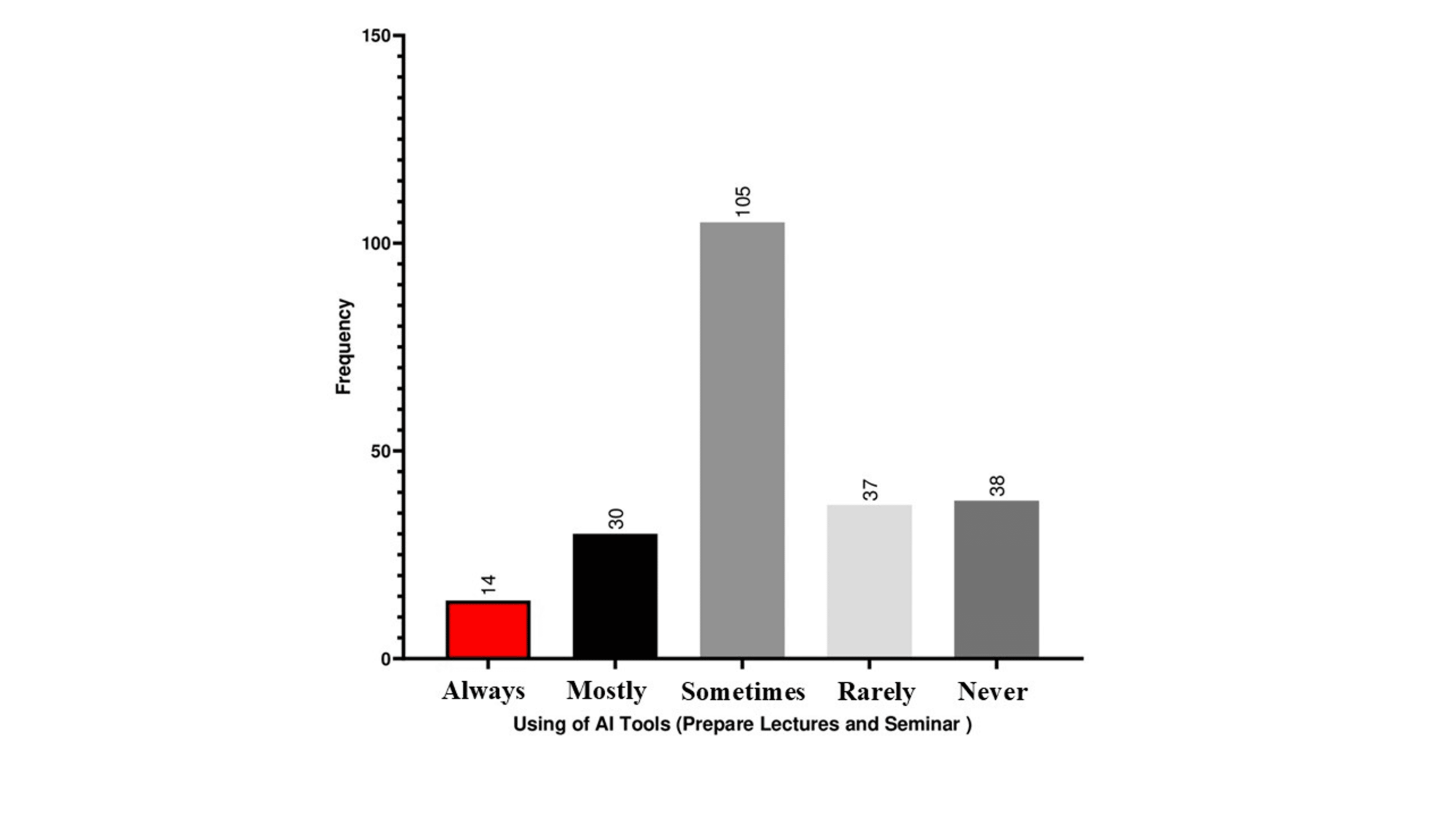
Students’ attitudes toward using AI tools for preparing lectures and seminars

Figure 1 shows that the most frequently given response was "sometimes," 105 (46.87%) indicating that most respondents only occasionally used AI tools to prepare lectures and seminars. Always, mostly, rarely, and never were the least common responses (14 (6.25%), 30 (13.39%), 37 (16.51), and 38 (16.96), respectively). This finding suggested that although AI technologies are used here, most medical students do not use them as their main approach.

## Discussion

The current study is the first to investigate medical students' attitudes and perceptions regarding the use of AI in the Kurdistan region of Iraq. A recent study showed that most participants (83%) used smartphones and tablets, while another study reported dissimilar results [14]. In addition, talking and messaging on a smartphone were the most common use cases, followed by browsing the internet and social media, while buying was the least common use case. Additionally, university students primarily utilize their smartphones when they are bored, alone, or waiting for someone [14]. In addition, on their smartphones, students have installed approximately 25 apps. Social media applications are the most popular, while utility-based and travel-related apps are the least helpful. With Facebook as their main social networking platform, students open the platform six times a day [15]. In addition, for higher education students, m-learning has swiftly emerged as the preferred modern method of learning and obtaining information to combine many learning modalities [16]. Medical students must teach themselves to use AI tools for various tasks in learning and conducting medical research, as these tools can provide insights that are easily understandable for students; however, students must use them carefully and supervise AI recommendations [17].

In the present study, a small proportion of the students believed that using manikin was a bad choice; thus, the students' perceived self-efficacy was unaffected by the manikin's death simulation [18]. For the purpose of training and education in health professions, virtual patients perform interactive computer simulations of actual clinical events. The virtual patients are designed to present with genuine symptoms, react to the interventions made by the students, and produce dynamic clinical scenarios. When gathering data and making recommendations for differential diagnosis, medical management, and patient follow-up, the learner adopts the role of a healthcare provider. These computer programs can mimic different medical conditions and expose trainees to difficulties they encounter in the real world [19,20]. Medical students can practice their clinical reasoning and communication skills by engaging with virtual patients, resulting in an immersive and dynamic virtual environment that replicates real-world circumstances [21].

Virtual reality (VR) can enhance the immersiveness of learning encounters with virtual patients. VR is a software-based technology that generates a three-dimensional virtual world [22,23]. VR creates a realistic-feeling computer-generated environment for the user through the use of glasses or a head-mounted display. However, augmented reality (AR) enhances the real world by superimposing virtual elements on a user's vision through the use of a smartphone or other device [24].

In the present study, most of the students, who were half of them, considered themselves experts in using AI. Previously, it was found that AI and tools related to AI are important for study and can provide important medical information, especially for students [21]. AI solutions that are connected with learning management systems (LMSs) give students the tools they need to master material at their own speed [25]. The learner's knowledge level is ascertained by computer algorithms, which then deliver tailored instructional information to help with content mastery. Learning events are effectively paced and sequenced by AI-based platforms, which then prompt learners to take targeted remediation actions. These individualized and flexible teaching strategies increase student effectiveness and efficiency [16,25].

In addition, AI-based resources could be helpful for radiology, pathology, nursing, and microbiology education. Content-based image retrieval (CBIR), which is utilized in radiology education and research, is an innovative technique. Using data gathered from the photos, CBIR assists by looking for images with contents comparable to a reference image [26].

In a similar vein, AI combined with machine learning is being used to identify microbial diseases and has enormous potential for education and training microbiology technicians [27]. The development of AI-based deep learning technologies that target cellular pictures, on the other hand, has the potential to revolutionize diagnostic pathology teaching [28].

A large percentage of students expressed concerns about depending only on manikins. Nevertheless, a somewhat larger group finds them useful as long as they are not the exclusive training approach. According to these findings, students still favor in-person patient contact for the development of comprehensive clinical skills, even though they acknowledge the benefit of manikins for certain learning objectives. A previous study showed that 17% of respondents said they would feel more at ease utilizing a manikin, whereas 58% said they would feel more at ease working with an actor [29].

Online surveys are convenient, cost-effective, and accessible, but they can have drawbacks, including poor response quality, limited question types, sample problems, response rate, and nonresponse bias. Those with poor online skills or access may not be able to answer due to technological or access concerns. Researchers are unable to clarify misunderstandings or go further into responses from online surveys. Compared to establishing rapport with face-to-face contacts, establishing rapport with respondents in online surveys is more difficult. Identity verification is not always possible; therefore, security and data privacy considerations are critical. Moreover, online survey environments are more difficult for researchers to manage, which may have an impact on responses and interpretations. Even with these drawbacks, online surveys are still useful for rapidly and effectively gathering data.

## Conclusions

While many agree that digital tools and simulations are useful teaching tools, they are frequently viewed as adjunctive approaches. Better integration and training are required for the infrequent use of AI tools in medical education. Students can receive thorough and efficient education through a blended learning strategy that combines technology and conventional approaches. For a thorough and successful learning experience, manikins and simulations should be used in conjunction with actual patient contacts and conventional laboratory work, even though they provide a safe setting. Further study is necessary to explore the positive and negative aspects of using AI in medical education.
